# Selenium nanoparticles-loaded chitosan/citrate complex and its protection against oxidative stress in d-galactose-induced aging mice

**DOI:** 10.1186/s12951-017-0324-z

**Published:** 2017-12-20

**Authors:** Kaikai Bai, Bihong Hong, Zhuan Hong, Jipeng Sun, Changsen Wang

**Affiliations:** 1 Third Institute of Oceanography, State Oceanic Administration, Xiamen, 361005 People’s Republic of China; 2 Engineering Research Center of Marine Biological Resource Comprehensive Utilization, State Oceanic Administration, Xiamen, 361005 People’s Republic of China

**Keywords:** Selenium, Nano, Chitosan, Citrate, Oxidative stress

## Abstract

**Background:**

Selenium (Se) is an indispensable trace element required for animals and humans, and extra Se-supplement is necessary, especially for those having Se deficiency. Recently, selenium nanoparticles (SeNPs), as a special form of Se supplement, have attracted worldwide attention due to their distinguished properties and excellent bioactivities. In this present study, an eco-friendly and economic way to prepare stable SeNPs was introduced. SeNPs were synthesized in the presence of chitosan (CTS) and then embedded into chitosan/citrate gel, generating selenium nanoparticles-loaded chitosan/citrate complex (SeNPs-C/C). Additionally, the clinical potential of SeNPs-C/C was evaluated by using d-galactose (d-gal)-induced aging mice model.

**Results:**

SeNPs in high uniform with an average diameter of around 50 nm were synthesized in the presence of chitosan, and reversible ionic gelation between chitosan and citrate was utilized to load SeNPs. Subsphaeroidal SeNPs-C/C microspheres of 1–30 μm were obtained by spay-drying. Single SeNPs were physically separated and embedded inside SeNPs-C/C microparticles, with excellent stability and acceptable release. Acute fetal test showed SeNPs-C/C was safer than selenite, with a median lethal dose (LD_50_) of approximately 4-fold to 11-fold of that of selenite. Oral administration of SeNPs-C/C remarkably retarded the oxidative stress of d-gal in Kunming mice by enhancing the activity of antioxidase, as evidenced by its significant protection of the growth, liver, Se retention and antioxidant bio-markers of mice against d-gal.

**Conclusions:**

The design of SeNPs-C/C opens a new path for oral delivery of SeNPs with excellent stability, energy-conservation and environment-friendliness. SeNPs-C/C, as a novel supplement of Se, could be further developed to defend the aging process induced by d-gal.
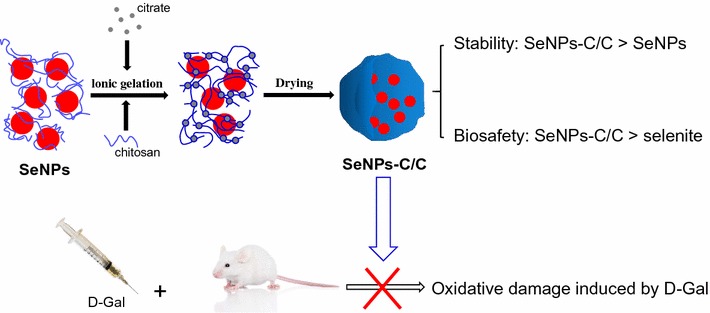

## Background

As well known for its photoelectric and semiconductor properties, Selenium (Se) has been widely applied in many fields such as solar cells, rectifiers, photographic exposure meters and xerography [1]. Meanwhile, Se is an indispensable trace element required for animals and human being [2]. It plays an essential role in preventing various diseases such as cardiovascular disease, hypercholesterolemia and certain cancers [2]. To meat the daily requirement of Se, extra Se supplementation is needed, especially for those suffering from Se deficiency [2]. Some Se compounds in organic or inorganic form, have been used for decades to avoid selenium deficiency in animals or human beings [2]. But these Se supplements, especially the inorganic ones, have to face with a very narrow margin between their nutritional dosage and their toxicity [2–4].

Recently, selenium nanoparticles (SeNPs), a unique type of elemental selenium of nano defined size with bright red appearance, have aroused worldwide attention due to their distinguished properties and excellent biological activities [2–4]. It is able to scavenge free radicals in vitro [5] and to improve growth performance, serum oxidant status and Se retention in vivo [6, 7]. Amazingly, compared with other Se compounds such as selenite [4, 7], selenomethionine [3], Se-yeast [7] and Se-methylselenocysteine [8], SeNPs exhibit much lower acute toxicity while increasing the activities of selenoenzymes. In addition, SeNPs can inhibit the growth of microorganisms [9], and it also exhibits antitumor activities both in vivo [10, 11] and in vitro [12]. Therefore SeNPs are regarded as a prospective Se formulation due to its potential in nutritional supplement use, chemoprevention and chemical therapy against cancer.

However, the application of SeNPs is restrained by the following facts: (1) economic and environment-friendly ways to synthesize small SeNPs are needed [13–15] and (2) bare SeNPs usually enlarge, aggregate and finally transform into gray/black analog that is thermodynamically stable, but biologically inert [16, 17]. Both of the problems are related to the final size and stability of SeNPs, on which the activities of SeNPs are dependent. Likely an increase in the size of SeNPs results in reduction in these nanoparticles’ biological activities (e.g., scavenging multiple radical species [5], enhancing Se retention [18], and up-regulating GSH S-transferase activity [18]). It seems only SeNPs with a smaller size and good stability can guarantee high bioactivities. To address these problems, some compounds including polysaccharides [17, 19, 20], monosaccharides [21], proteins [4, 22–24], animo acids [25], polyphenols [26], melatonin [27], ATP [28], plant extract [11, 29] and even the culture of microorganism [9], had been utilized to synthesize and stabilize SeNPs. Among them, polysaccharide is considered as an appropriate template for fabricating SeNPs when considering energy efficiency and eco-friendliness [17]. But the SeNPs synthesized by using polysaccharides were still suffering from unacceptable instability, due to enlargement of size and decline of zeta potential during preservation in aqueous environment [17, 20]. Thus, a new way to prepare stable SeNPs is needed.

Aging is a time-dependent decline process in physiological function of the organism, involving many physiological dysfunctions such as fertility decrease and increasing susceptibility to endogenous and external threats [30]. Being able to increase the production of reactive oxygen species (ROS), oxidative stress plays a key role in the process of biological aging [31]. Various oxidative damages in animals and humans could be retarded by Se compounds [2–8, 30]. However, the study reporting the effect of SeNPs on the aging oxidative stress was rather poor. The application of SeNPs could be extensively extended if it was able to defend against the aging oxidative stress with excellent stability and advantageous bio-safety.

Chitosan (CTS) is the only positively charged natural polysaccharide, possessing excellent biocompatible and biodegradable properties [32]. It has been extensively studied in the pharmaceutical industry for its potential in the development of medicine delivery system [32]. In this study, SeNPs were synthesized in the presence of CTS and the reversible ionic gelation [33, 34] between CTS and citrate was applied to embed SeNPs, giving birth to selenium nanoparticles-loaded chitosan/citrate complex (SeNPs-C/C). It was expected each SeNP would be physically separated and embedded into solid SeNPs-C/C, with acceptable stability and good release. Additionally, the bio-safety of SeNPs-C/C was studied and the antioxidant activities of SeNPs-C/C against the oxidative stress induced by d-galactose (d-gal) were investigated, in order to evaluate the potential of SeNPs-C/C in clinical application.

## Results and discussion

### Properties of SeNPs

**Scheme 1:**
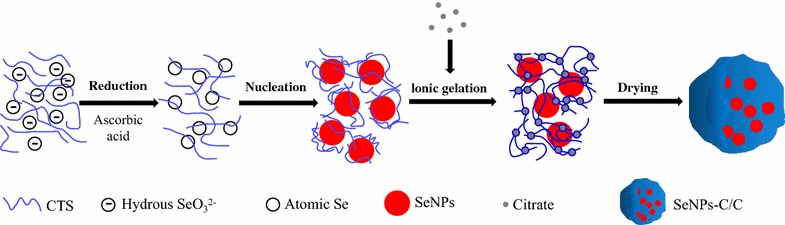
The preparation process of SeNPs-C/C. Briefly, CTS and Vc were dissolved in acetic acid solution. Then, aqueous selenite was slowly added to the CTS/Vc solution, resulting in atomic Se. Atomic Se nucleated to form Se nucleus, and the Se nucleus assembled into SeNPs. Consequently, the mixture of aqueous CTS and SeNPs was added to citrate solution, generating SeNPs-C/C. The wet SeNPs-C/C was shredded and then spay-dried to obtain solid SeNPs-C/C microparticles

**Fig. 1:**
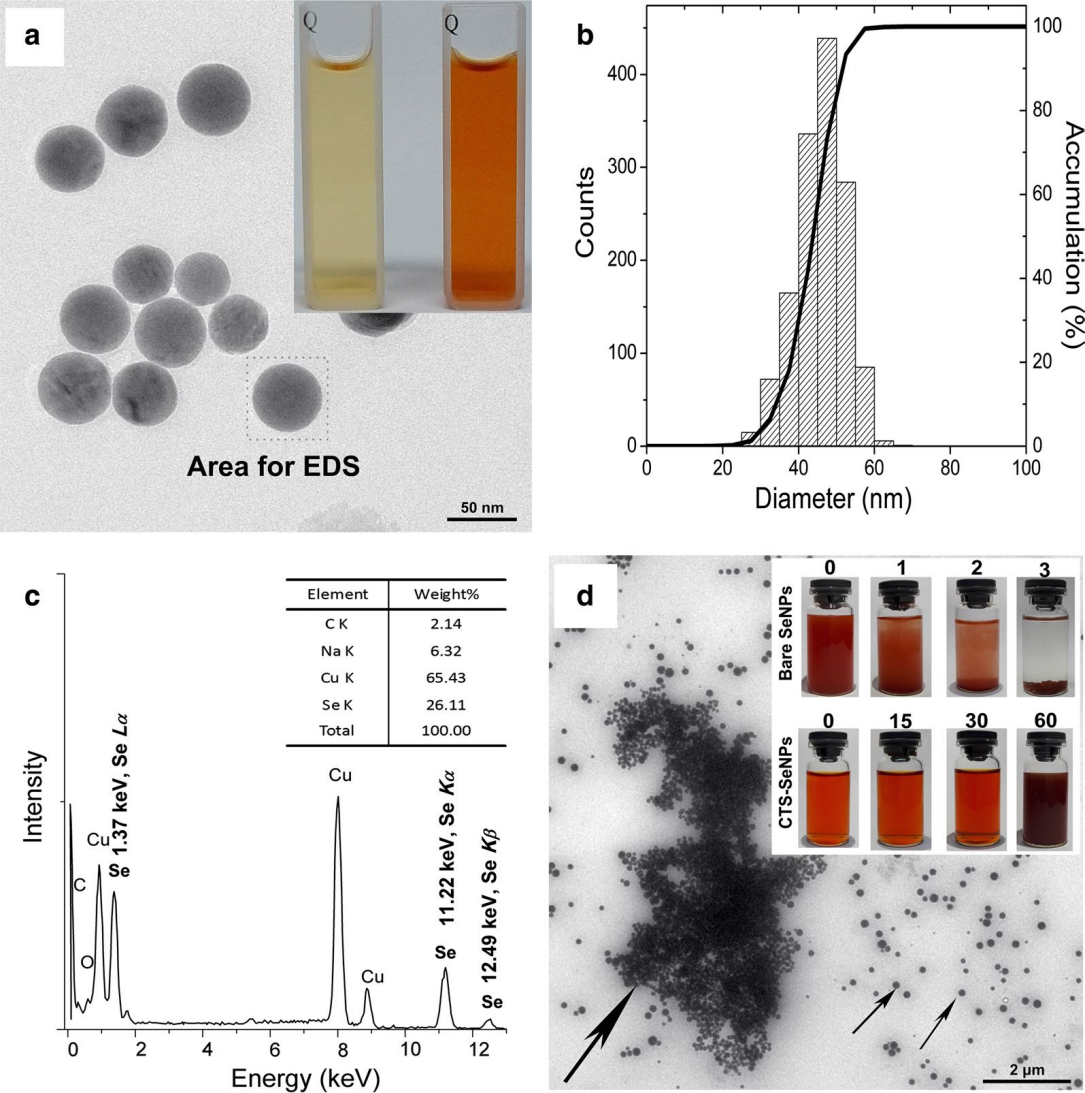
Morphology and formation of SeNPs freshly synthesized in the presence of aqueous CTS. **a** TEM image of CTS-SeNPs and their appearance (inset). **b** The size distribution of CTS-SeNPs measured basing on TEM results. **c** Typical EDS spectra of CTS-SeNPs and their elemental composition. **d** TEM image of CTS-SeNPs after a 60-days preservation and photos of SeNPs in the absence or presence of CTS during storage at 25 °C (inset). Big arrows represent aggregated SeNPs while smaller ones represent free SeNPs

Some polysaccharides [17, 19, 20] had been utilized to synthesize and stabilize SeNPs. Thus, SeNPs synthesized was of various morphology, such as amorphous, sphere, wire, rob and tube [16, 17, 20, 35]. Herein, aqueous Se(IV) was chemically reduced by ascorbic acid (Vc) to synthesize SeNPs (Se^0^) in the presence of CTS. The initial colloid nucleated and then assembled into SeNPs (Scheme 1). Consequently, monodisperse spherical CTS-SeNPs in high uniform was obtained, with red appearance (Fig. 1a). The SeNP cores could be unequivocally identified as the organic shell around these cores provided at best low contrast in transmission electron microscopy (TEM) images [36]. The mean size of SeNPs cores was measured to be around 50 nm (Fig. 1b). Besides, typical Se peaks (1.37, 11.22 and 12.49 keV) were found in energy dispersive X-ray spectroscopy (EDS) spectra of CTS-SeNPs, strongly confirming the elemental nature of these nanoparticles (Fig. 1c).

Bare SeNPs synthesized in absence of CTS quickly agglomerated in few days as shown in Fig. 1d inset, but CTS-SeNPs could be stable for at least 30 days. It suggested CTS can control the size and stability of SeNPs. However, the stability of SeNPs could not last for over 60 days even in the presence of CTS, as evidenced by the typical agglomeration of SeNPs shown in Fig. 1d. Similar instability was reported by Kong [20] who aimed to stabilize SeNPs with gum arabic, and it was also observed by Yu [37]. Zhang [17] pointed out the decrease of SeNPs’ zeta-potential in aqueous circumstance was unavoidable during storage, ant it occurred in a time-dependent manner. Considering the relationship between nanoparticles’ stability and their zeta-potential, longer preservation of SeNPs in aqueous CTS might be a challenge though CTS was an ideal polysaccharide to fabricate these nanospheres.

### Characterization of SeNPs-C/C

#### Morphology and formation

Both bare SeNPs and CTS-SeNPs were unavailable for commercial application in oral administration systems due to their awful stability. To address this problem, SeNPs were supposed to be preserved in solid state. As shown in Scheme 1, the ionic gelation [33, 34] between CTS and citrate was introduced to embed SeNPs into the physically cross-linked CTS/citrate gel. The moisture of the gel was evaporated to obtain solid SeNPs-C/C with red appearance. The Se content of SeNPs-C/C was determined by utilizing inductively coupled plasma mass spectrometry (ICP-MS) [38] and it could be adjusted by modifying material ratio. A SeNPs-C/C sample (10.5 g Se kg^−1^) was selected to conduct the rest of the study.

**Fig. 2:**
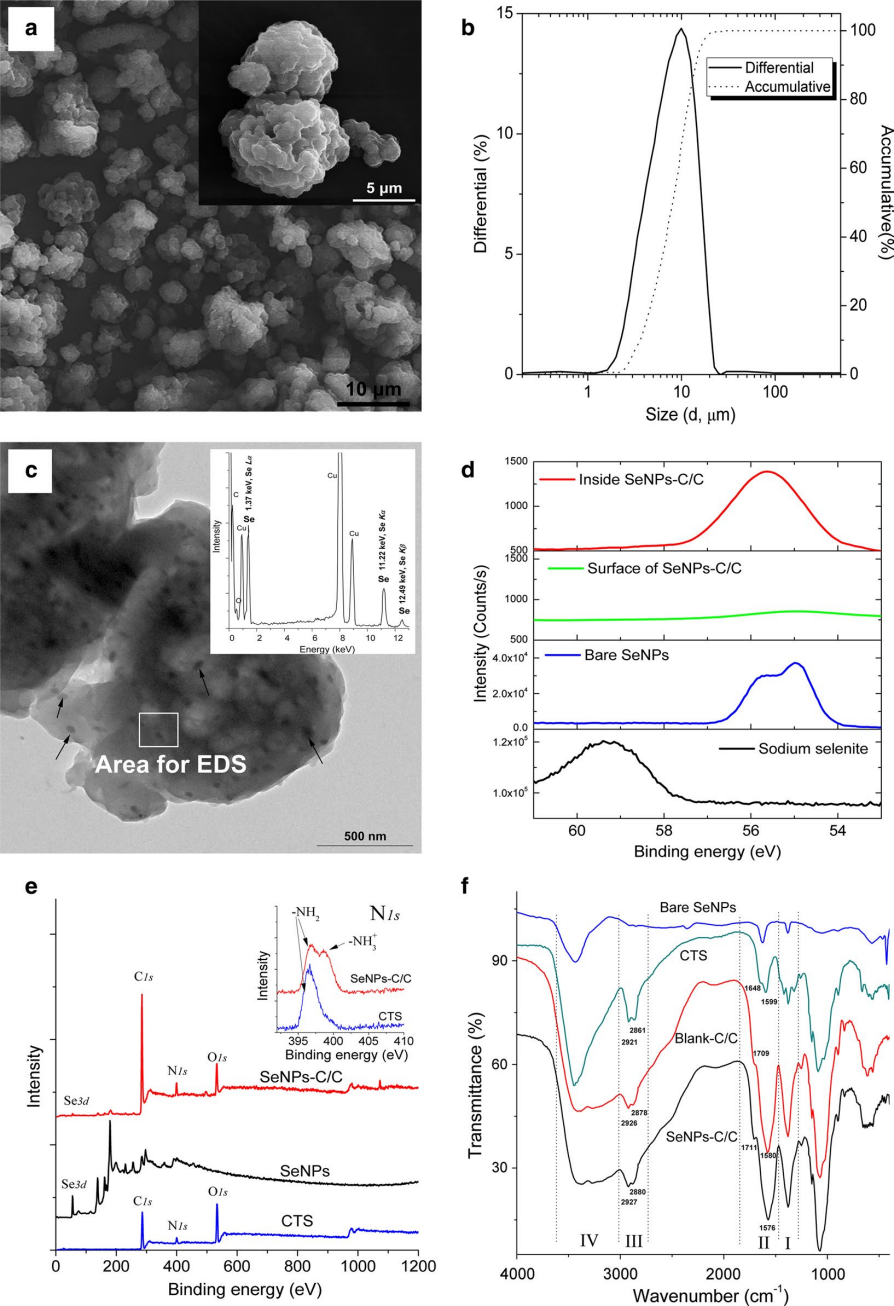
Characterization of SeNPs-C/C. **a** SEM image of SeNPs-C/C and inset for the details. **b** Size distribution of SeNPs-C/C measured by Mie theory. **c** TEM image of SeNPs-C/C and inset for EDS spectra, with arrows indicating single SeNPs embedded into SeNPs-C/C. **d** Se *3d* XPS pattern and **e** wide-range XPS pattern of SeNPs-C/C with inset for N*1s* XPS result, recorded by a high-resolution photoelectron spectrograph (Escalab 250Xi) equipped with a monochromatic Al Kα X-ray source. Argon ion etching lasting for 60 s was applied to expose Se information inside SeNPs-C/C. **f** FTIR spectra of bare SeNPs, CTS, Blank C/C and SeNPs-C/C

Scanning electron microscopy (SEM) images shown in Fig. 2a indicated SeNPs-C/C was a collection of various microparticles with irregularly wrinkled surface. Some of SeNPs-C/C might get together as presented in Fig. 2a inset. The size of these microparticles was 1–30 μm as shown in Fig. 2b, determined by size distribution analysis basing on Mie scattering theory [39]. As expected, individual SeNPs were clearly observed in the TEM image of SeNPs-C/C (Fig. 2c). Typical EDS spectra of Se were also found in the area containing dark nanoparticles (Fig. 2c inset). Apparently, single SeNPs dispersed in solid SeNPs-C/C microparticles.

#### XPS results

X-ray photoelectron spectroscopy (XPS) patterns of SeNPs-C/C were recorded to explore more details of SeNPs-C/C. Typical Se *3d* peaks of Se(0) and Se(IV) were detected at 55.3 and 59.5 eV, respectively (Fig. 2d), confirming Se in SeNPs-C/C was in elementary status. However, the Se *3d* signals on the surface of SeNPs-C/C was remarkably weaker than that inside SeNPs-C/C since argon ion etching might expose the SeNPs inside. It implied most of the SeNPs were enclosed inside SeNPs-C/C. Additionally, the characteristic peaks of –NH_3_^+^ and –NH_2_ displayed clear variation between CTS/citrate complex and CTS (Fig. 2e inset). It was consistent with the fact that CTS was positively charged in the case of ionic cross-linked CTS [33, 34].

#### FTIR results

The intermolecular interaction of samples was characterized by Fourier transform infrared spectroscopy (FTIR) (Fig. 2f). Some characterization peaks of CTS, observed at 3449, 1648, 1599, 1422, 1381, and 1030 cm^−1^, were regarded as O–H or N–H stretch, C=O stretching from amide I, N–H bending and C–N stretching from amide II, –CH_2_ bending, –CH_3_ symmetrical deformation, and skeletal vibration of C–O stretching, respectively. But the spectrum of SeNPs-C/C was different, highlighted in the wavenumber ranging from 1000 to 3700 cm^−1^. The peak of O–H or N–H stretch became wider and flatter, indicating that hydrogen bonding was enhanced (Zone IV) [40]. The newborn peak at around 1710 cm^−1^ was attributed to the carboxyl groups from citrate (Zone II). The peaks of amide I and amide II in the blank chitosan/citrate complex (Blank-C/C, free of SeNPs) overlapped and became an intensive absorption peak at 1576 cm^−1^, due to the electrostatic interaction between amino groups of CTS and carboxyl groups of citrate (Zone II) [34]. It was in accordance with the N_1s_ XPS results presented in Fig. 2e inset. Besides, the left-shift of C–H stretch peaks at 2927 and 2880 cm^−1^ as compared with CTS implied SeNPs-C/C is less crystalline than CTS (Zone III) [41]. Nonetheless, no significant difference could be found between SeNPs-C/C and Blank-C/C. Probably the signal of Se was too weak or/and Se was rather poor.

### Release of SeNPs from SeNPs-C/C

**Fig. 3:**
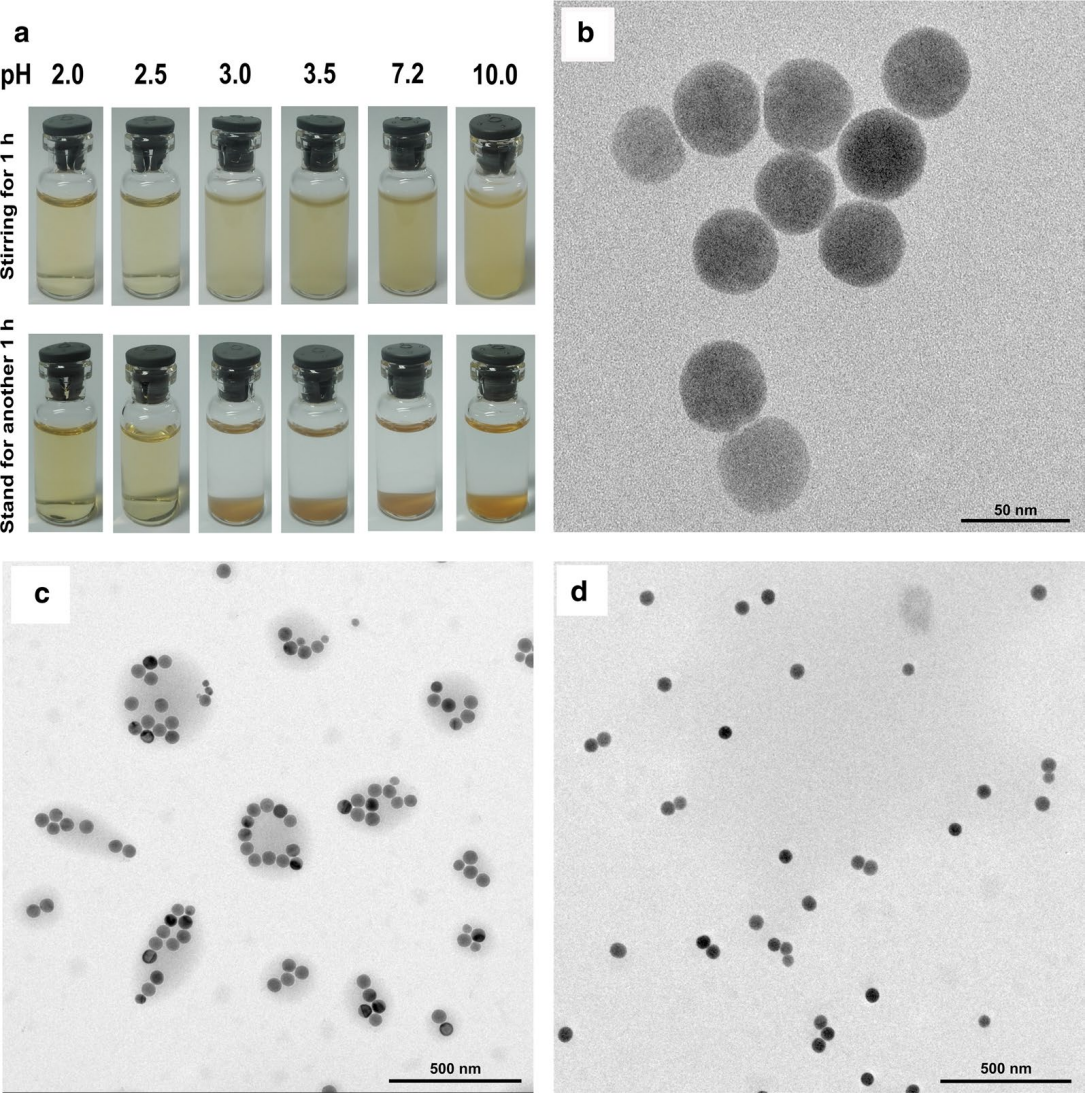
Release of SeNPs from SeNPs-C/C. **a** Photos taken when SeNPs were mixed with HCl or NaOH solution of pH 2.0, 2.5, 3.0, 3.5, 7.2 and 10.0. Photos were taken at 1 h after the mixing. After standing for another 1 h, the solutions were photoed again. **b** TEM image of SeNPs obtained at 8 h after mixing SeNPs-C/C with pH 2.5 HCl solution. TEM images of SeNPs taken at **c** 30 min or **d** 4 h after adding SeNPs-C/C to HCl solution (pH 2.5)

The SeNPs embedded in SeNPs-C/C should be released in the digestive intact to display its effects on body. In this experiment, SeNPs-C/C was treated with HCl or NaOH solution of different pH to study the release of SeNPs. As shown in Fig. 3a, SeNPs-C/C could be absolutely dissolved in HCl solution (pH 2.0–2.5) by modulate stirring for 30 min, without any visible precipitation. Nonetheless, solid SeNPs-C/C could not disappear when pH value exceeded 3.0, even under fierce stirring for 8 h. In addition, intact SeNPs could be easily observed by TEM after the dissolution of SeNPs-C/C in HCl solution (pH 2.0–2.5) (Fig. 3b). It suggested SeNPs can be released from SeNPs-C/C in stomach of animals and human beings. Because the pH in mammalian stomach could be low as 1–3 [42].

The results could be explained by the fact that the ionic gelation of CTS/citrate was dependent on the environmental pH. To be more specific, the ionization of citrate could be extremely different in the case of pH 2.0–10.0 as the pKa values of citrate are 3.1, 4.7 and 6.4 [43]. When the pH was of far less than 3.1, citrate could not be ionized at all, resulting in the collapse of the ionic cross-linking between CTS and citrate.

Moreover, the process of collapse was studied by examining the TEM images of the released SeNPs, of which were obtained at different time points. As presented in Fig. 3c, many gray shadows containing SeNPs were observed at 30 min after the addition of SeNPs-C/C to HCl solution (pH 2.5). It was SeNPs that were surrounded by organic substance, probably the incompletely dissolved CTS/citrate. At 4 h after mixing SeNPs-C/C with HCl solution, a great number of free SeNPs, however, were found in the TEM image without any agglomeration (Fig. 3d). It implied SeNPs are quickly released from SeNPs-C/C and they can keep intact in acid condition for hours. It was likely the dissolved CTS can still guarantee their stability. Possibly the surface of the released SeNPs might be covered by protein corona [36, 44] in digest tract if orally administrated, which could also be beneficial to the stability. Based on the results above, the design of SeNPs-C/C allowed the oral administration of SeNPs with good stability and favorable release rate.

### Stability of SeNPs-C/C

The stability of SeNPs-C/C in storage was evaluated by stress testing [45] and accelerated testing [46]. In stress testing, the responses of SeNPs-C/C to high temperature, high humidity and strong light were studied. As shown in Table 1, SeNPs-C/C was very stable when preserved in the environment of 60 ± 1 °C, 80 ± 5% relative humidity (RH) and 5000 ± 500 Lx, with little change in terms of Se content and Se *3d* XPS spectra. In accelerated testing, excellent stability was still guaranteed after 6 months of storage in a simulated package environment (40 ± 2 °C, 75 ± 5% RH, dark). As expected, each sample remained its original appearance during these experiments, and SeNPs embedded were easily released (data not shown). The results implied the stability of SeNPs-C/C can meat the standard for common food storage and food transportation [45, 46].

**Table 1:** The stability of SeNPs-C/C against temperature, humidity and light (n = 3)

Test type	Storage condition^a^	Storage time	Se content (mg kg^−1^)	Se *3d* peak (eV)
No test	–	0 day	10.6 ± 0.3	55.2
Stress testing	60 ± 1 °C, 40 ± 5% RH, dark	10 day	11.0 ± 0.2	55.3
	25 ± 1 °C, 80 ± 5% RH, dark	10 day	10.5 ± 0.8	55.3
	25 ± 1 °C, 40 ± 5% RH, 5000 ± 500 Lx	10 day	10.6 ± 0.1	55.3
Accelerated testing	40 ± 2 °C, 75 ± 5% RH, dark	1 month	10.7 ± 0.2	55.3
		2 month	10.6 ± 0.4	55.2
		3 month	10.7 ± 0.3	55.2
		6 month	10.2 ± 0.2	55.2

^a^After individual treatment, each sample was dried to constant weight at 60 °C before Se content determination and XPS Se *3d* scanning

RH, relative humidity

### Acute toxicity of SeNPs-C/C

In the acute lethal test, SeNPs-C/C and sodium selenite at increasing doses were orally administered by single gastric administration, and the mortality was recorded within 14 days. The results presented in Table 2 illustrated selenite was very dangerous to both Institute of Cancer Research (ICR) mice and Kunming (KM) mice, with a median lethal dose (or LD_50_) of 3.4 and 8.8 mg Se kg^−1^ body weight (bw), respectively. However, the LD_50_ of SeNPs-C/C against ICR mice and KM mice were 39.1 and 37.2 mg Se kg^−1^ bw, respectively. It suggested the acute toxicity of SeNPs-C/C is only 1/11 or 1/4 of that of selenite based on Se dose. Considering the excellent safety of Blank-C/C with a LD_50_ over 15 g kg^−1^ bw and the quick release of SeNPs in acid environment, it was SeNPs within SeNPs-C/C that were responsible for the acute toxicity.

**Table 2:** Acute lethal results by single oral administration in mice (n = 10)

Sample	Se content (mg g^−1^)	ICR mice		KM mice	
		LD_50_ (g kg^−1^ bw)	LD_50_(Se) (mg Se kg^−1^ bw)	LD_50_ (g kg^−1^ bw)	LD_50_(Se) (mg Se kg^−1^ bw)
Sodium selenite	456.7	7.4 × 10^−3^ (5.3–10.3) × 10^−3a^	3.4 (2.4–4.7)^b^	1.92 × 10^−2^ (1.62–2.27) × 10^−2a^	8.8 (7.4–10.4)^b^
Blank-C/C	–	> 15	–	> 20	–
SeNPs-C/C	10.5	3.72 (2.57–5.40)^a^	39.1 (27.0–56.7)^b^	3.54 (2.97–4.23)^a^	37.2 (31.2–44.4)^b^

LD_50_ = median lethal dose; LD_50_(Se) = LD_50_ × Se content

^a^The LD_50_ of 95% confidence interval

^b^The LD_50_(Se) of 95% confidence interval

The results above were consistent with some previous studies reporting the seven-fold of bio-safety of BSA-based Nano-Se superior to that of selenite [4, 24]. There might be some similarities. Perhaps SeNPs loaded by polysaccharide or protein could exhibit similar acute toxicity if they possessed similar basic physicochemical properties such as shape, size, chemical composition and surface properties, since the physico-chemical properties of nanoparticles might contribute great to the toxicity [36, 44, 47]. However, the properties of SeNPs in vivo, such as the size and aggregation in gastrointestinal tract, had not been systematically studied yet. Also correlating the nanoparticles’ properties to their toxicological response was a challenge [36, 44]. Thus it needed more investigation, though it was clear that SeNPs in the form of SeNPs-C/C were much safer than selenite.

### Protecting effects of SeNPs-C/C against aging damage induced by d-gal

d-Gal can be metabolized at normal concentration. But when at high levels, it can be converted into aldose and hydroperoxide under the catalysis of galactose oxidase [48, 49]. The metabolites might generate a superoxide anion and oxygen-derived free radicals (OFR), resulting in aging-like oxidative damage to body [48, 49]. Herein, aging mice model induced by d-gal was established, and the potential of SeNPs-C/C as a protective agent against aging oxidation was evaluated.

#### Growth inhibition

**Fig. 4:**
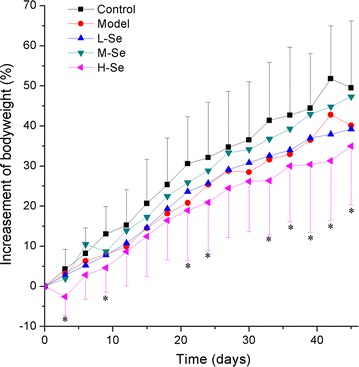
The body weight (bw) of KM mice during the 45-days d-gal injection experiment. The details of administration in each group are presented in Table 3. In brief, the daily administrations were: Control: water, Model: d-gal (100 mg kg^−1^ bw) + water, L-Se: d-gal (100 mg kg^−1^ bw) + SeNPs-C/C (15.8 mg kg^−1^ bw), M-Se: d-gal (100 mg kg^−1^ bw) + SeNPs-C/C (31.6 mg kg^−1^ bw) and H-Se: d-gal (100 mg kg^−1^ bw) + SeNPs-C/C (63.2 mg kg^−1^ bw). *P < 0.05 vs Control group

With the initial body weight of about 20 g, male KM mice were daily treated with saline (as control), d-gal (as model, 100 mg kg^−1^ bw) and SeNPs-C/C (15.8, 31.6, 63.2 mg kg^−1^ bw) + d-gal (100 mg kg^−1^ bw) (presented in Table 3). As shown in Fig. 4, the injection of d-gal led to less body weight as compared with control, consistent with other reports [30, 50]. Low dose (15.8 mg kg^−1^ bw) of SeNPs-C/C was unable to change the tendency. But SeNPs-C/C at a dose of 31.6 mg kg^−1^ bw retarded the growth inhibition caused by d-gal, resulting in insignificant body weight loss as compared with control. However, a high dose (e.g., 63.2 mg kg^−1^ bw) of SeNPs-C/C inhibited mice’ growth instead of reducing the growth inhibition generated by d-gal, even resulting in unexpected loss of body weight (*P* < 0.05 vs control). It could be explained by two facts: (1) Se compounds show toxicity when taken above their nutritional dosage, [3, 4] and (2) nanotoxicity of inorganic nanoparticles can be generally triggered in two ways, either due to reaction of their surface with the environment or by leaching toxic ions, which in both cases leads to the production of ROS [36]. Optimal dose of SeNPs-C/C might alleviate the d-gal-induced growth inhibition.

**Table 3:** The daily administration during the d-galactose oxidative experiment (KM mice)

Group	n	Injection	Oral administration
Control	10	None	Sterile water
Model	15	d-Gal (100 mg kg^−1^ bw)	Sterile water
L-Se	15	d-Gal (100 mg kg^−1^ bw)	SeNPs-C/C (15.8 mg kg^−1^ bw)
M-Se	15	d-Gal (100 mg kg^−1^ bw)	SeNPs-C/C (31.6 mg kg^−1^ bw)
H-Se	15	d-Gal (100 mg kg^−1^ bw)	SeNPs-C/C (63.3 mg kg^−1^ bw)

bw, body weight

#### Tissue protection by SeNPs-C/C

**Fig. 5:**
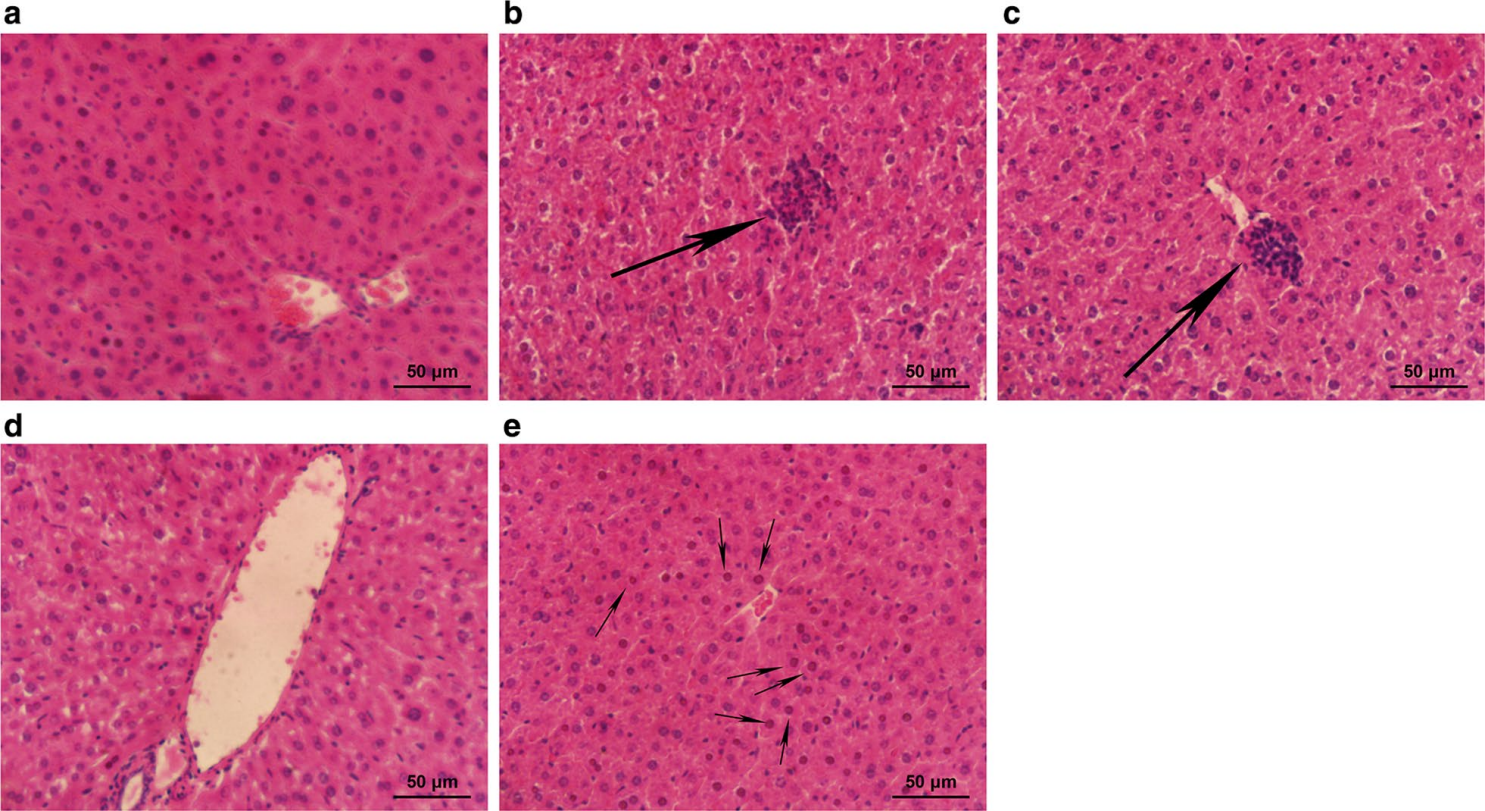
Histological details of liver sections in different groups of mice: **a** Control, **b** Model, **c** L-Se, **d** M-Se and **e** H-Se, obtained by using HE staining. The daily administrations are presented in Table 3, lasting for 45 days. After the last administration, mice were sacrificed to obtain serum and liver. Big arrows refer to congregated leucocytes and migratory leucocytes, while the smaller ones represent necrosis

The impacts of SeNPs-C/C on the tissues of d-gal-treated KM mice were investigated for more details. The injection of d-gal significantly increased the relative weigh of thymus (Model: 3.3 ± 0.6 g kg^−1^ vs Control: 2.2 ± 0.6 g kg^−1^, *P* < 0.05) and spleen (Model: 5.3 ± 1.0 g kg^−1^ vs Control: 3.7 ± 1.0 g kg^−1^, *P* < 0.05), while oral administration of SeNPs-C/C did not have clear impact on relative liver weight. However, difference could be found among these five groups in terms of liver section. Rare damaged cells could be seen in control group (Fig. 5a). d-Gal treatment (100 mg kg^−1^ day^−1^) caused visible histology changes including structure damage, cellular swelling, necrosis and leucocyte infiltration in mice livers (Fig. 5b), which was very common as described by Zhang [51] and Lei [52]. But SeNPs-C/C at doses of 15.8–31.6 mg kg^−1^ bw could alleviate the liver damage (Fig. 5c, d). Amazingly, little difference could be observed in liver’s physiological status between the M-Se group and control group (Fig. 5d vs a). SeNPs-C/C at a high dose (63.2 mg kg^−1^ bw) seemed to be helpless to reserve the destruction induced by d-gal, as spotty necrosis could still be seen (Fig. 5e). The histopathological results suggested SeNPs-C/C possess hepatoprotection activity against d-gal-induced liver injury.

#### Se retention enhanced by SeNPs-C/C

**Fig. 6:**
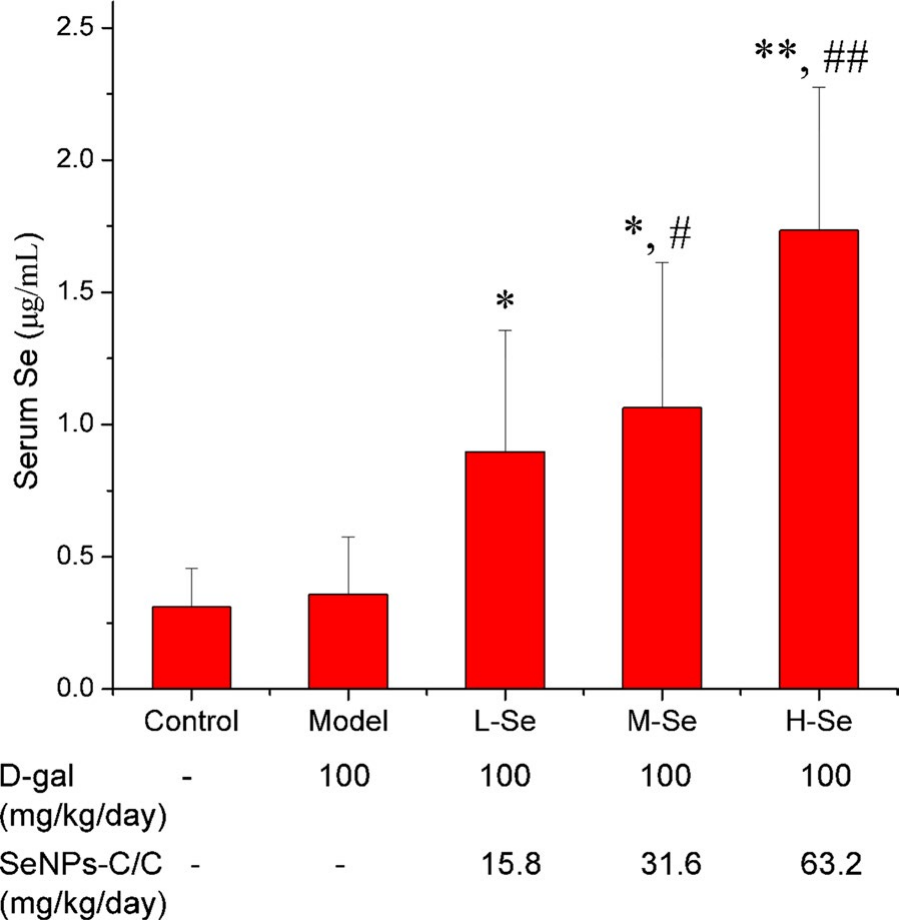
The serum Se contents of mice in different groups: Control, Model, L-Se, M-Se and H-Se. The daily administration of each group is shown in Table 3. After the last administration, mice were sacrificed to obtain serum and liver. The Se concentration of serum was determined by using ICP-MS assay. *P < 0.05 and **P < 0.01 vs Control group. ^#^ *P* < 0.05 and ^##^ *P* < 0.01 vs Model group

The Se level in body was monitored to study the Se-supplement ability of SeNPs-C/C in mice. The injection of d-gal had little influence on the serum Se content (Fig. 6). Oral administration of SeNPs-C/C, however, enhanced the Se retention in a dose-dependent manner. Similar results were found in other forms of Se, such as selenite [6], BSA Nano-Se [3, 6] and selenomethionine [3]. Considering the low Se content of the feed (< 0.1 mg Se kg^−1^ diet) and the estimable daily intake of adult KM mice (about 4–8 g diet day^−1^ each) [53], SeNPs-C/C was actually the main Se supplement. That was, SeNPs in SeNPs-C/C contributed to the Se retention within mice.

Some points about the Se retention of SeNPs-C/C should be stated. One was the determination of Se. Given that the average diameter of the dosed SeNPs was around 50 nm and that the serum concentration of Se was approximately 0.2–2.0 μg mL^−1^, the number density, however, was too low for any successful imaging of SeNPs by TEM [54]. Besides, the amount of serum SeNPs could not represent total Se level since SeNPs must undergo its absorption, distribution, metabolism and excretion (ADME) in animals and human beings. Thus, only the data of serum Se was presented here. Another point was that hyper-concentration of serum Se could be easily achieved by administration of overdose of SeNPs-C/C. But too high dose of Se-supplement might caused severe damage to body [3, 4, 30]. It was relative to some negative effects of SeNPs-C/C on KM mice, such as growth inhibition and liver damage.

#### Antioxidant activities of SeNPs-C/C

It is well known that Se plays an essential role in protecting animals and humans from oxidant damage by affecting the levels of antioxidant substrates and the activities of antioxidant enzymes [2–4]. Herein, oxidative stress was induced by 100 mg kg^−1^ bw of d-gal, as evidenced by the significant increase of thiobarbituric acid-reactive substances (TBARS, a kind of byproducts of lipid peroxidation) and decrease of glutathione (GSH), glutathione peroxidase (GSH-Px, a family of enzymes reduce lipid hydroperoxides to their corresponding alcohols and also reduce free hydrogen peroxide to water), superoxide dismutase [SOD, an enzyme family alternately catalyzes the dismutation (or partitioning) of the superoxide radical into either ordinary molecular oxygen or hydrogen peroxide] and catalase (CAT, an enzyme catalyzes the decomposition of hydrogen peroxide to water and oxygen) (*P* < 0.05 vs Control, shown in Table 4). Apparently, d-gal caused severe damage to mice’ antioxidant system, which was in accordance with many studies [30, 48–52]. However, the oxidative stress were greatly retarded by SeNPs-C/C at the dose of 15.8 or 31.6 mg kg^−1^ bw. Both L-Se and M-Se groups exhibited higher levels of GSH-Px, SOD, CAT and GSH and lower level of TBARS, as compared with model group (*P* < 0.01 or *P* < 0.001 vs Model). Amazingly, the activities of GSH-Px and SOD were fully recovered by SeNPs-C/C to normal levels, and stronger activity of CAT was found in L-Se and M-Se groups when comparing with model. The results above supported that SeNPs-C/C could effectively defend the d-gal-induced aging by enhancing the activities of antioxidant enzymes in vivo.

**Table 4:** The serum antioxidant activities of KM mice treated with d-gal and SeNPs-C/C

Group	GSH (mg L^−1^)	TBARS (nmol mL^−1^)	GSH-Px (U mL^−1^)	SOD (U mL^−1^)	CAT (U mL^−1^)
Control	19.14 ± 1.32	0.70 ± 0.04	22.67 ± 5.04	671.19 ± 47.47	9.67 ± 3.96
Model	5.34 ± 3.23^#^	0.95 ± 0.37^#^	16.84 ± 7.03^#^	459.23 ± 180.74^#^	5.91 ± 2.84^#^
L-Se	10.03 ± 6.10	0.60 ± 0.11**	26.21 ± 8.01**	671.21 ± 46.28***	16.12 ± 4.44***
M-Se	9.46 ± 3.80*	0.56 ± 0.09***	25.46 ± 3.65***	667.57 ± 41.46***	14.34 ± 6.88*
H-Se	7.76 ± 3.71	0.72 ± 0.36	16.64 ± 10.91	616.03 ± 102.83**	2.62 ± 1.78**

^#^ *P* < 0.05, vs Control

* *P* < 0.05, ** *P* < 0.01, *** *P* < 0.001, vs Model

Although SeNPS-C/C at optimal doses exhibited powerful protection against d-gal-induced oxidative stress, overdose of SeNPs-C/C (63.2 mg kg^−1^ bw) had to face with the rapid decline of antioxidase’s activity (Table 4). It suggested overdose of SeNPs-C/C is not longer helpful to recover the antioxidant system. That was consist with the negative impacts of SeNPs-C/C (63.2 mg kg^−1^ bw) on growth (Fig. 4) and liver (Fig. 5). The toxicity of SeNPs-C/C at a dose over 63.2 mg kg^−1^ bw might be systematic. Possibly some systematic damages triggered by nanoparticles (e.g., ROS [31, 36]) could affect the normal utilization of SeNPs-C/C in organism.

The ability of SeNPs-C/C to boost GSH-Px was worthy more attention in the aging experiment. Because a great number of studies have showed that GSH-Px activity in body decline with the increasing of age [30, 55, 56]. Moreover, the best way to scavenge ROS, according to Rowntree [57], is to remove potential damage of lipid hydroperoxides and hydrogen peroxide through the GSH-Px family of enzymes. Various forms of Se, such as selenite [7], selenium dioxide [30] and seleno-β-lactoglobulin [30], are helpful to improving GSH-Px activity, based on that Se can incorporate at least 25 human selenoproteins and enzymes as selenocysteine [2]. SeNPs-C/C significantly improved serum Se retention and boosted GSH-Px activity in d-gal-treated mice, confirming that Se in the formulation of SeNPs-C/C might be an efficacy agent to treat aging process induced by d-gal.

## Conclusions

In this study, SeNPs were synthesized in the presence of CTS, and then the ionic gelation between CTS and citrate was utilized to load SeNPs, giving birth to SeNPs-C/C. SeNPs were physically embedded into the spray-dried SeNPs-C/C microparticles, with excellent stability and good release. SeNPs might defend against d-gal-induced aging process in mice by enhancing the growth, live status, Se retention and antioxidant enzymes’ activities, with much better safety as compared with selenite. The design of SeNPs-C/C opens a new path for oral delivery of SeNPs with excellent stability, energy-conservation and environment-friendliness. SeNPs-C/C, as a novel Se formulation designed for nutritional supplement use, deserves further development in clinical application due to its bio-safety and its anti-aging property.

## Experimental section

### Materials and animals

CTS (90.32% deacetylated, average molecular weight of 37 kDa) of food grade were purchased from Aoxin Pharmaceutical Co. Ltd. (Taizhou, People’s Republic of China). The assay kits for measuring TBARS, GSH, GSH-Px, SOD, CAT and protein were provided by Jiancheng Bio-engineering Institute (Nanjing, People’s Republic of China). d-Gal of high purity grade was obtained from Ameresco (Solon, OH, USA). Regents of food grade including acetic acid, sodium selenite, ascorbic acid (Vc) and sodium citrate, and other reagents of analytic grade, were supplied by commercial suppliers.

KM mice of specific-pathogen-free (SPF) grade, 8–10 weeks old, 18–22 g bw, were supplied by Laboratory Animal Center, Shenyang Pharmaceutical University (Shenyang, People’s Republic of China) with the License No. SCXK (Liaoning) 2010-0001. ICR mice of SPF grade, 4–5 weeks old, 18–22 g bw, were purchased from Laboratory Animal Center, Xiamen University (Xiamen, People’s Republic of China) with the license No. SCXK (Fujian) 2013-0001. Mice were housed in a standardized sterile animal room with controlled temperature (25 ± 2 °C) and humidity (50 ± 10%) and a 12-h light/dark cycle.

### Synthesis of SeNPs and preparation of SeNPs-C/C

SeNPs was prepared by reducing selenite according to Zhang [17] with limited modification. Briefly, 1 g of CTS and 0.8 g of Vc were completely dissolved in 100 mL of 1% (w/w) acetic acid to attain CTS/Vc solution. After that, 5 mL of selenite aqueous solution containing 0.2 g of sodium selenite was dropwisely added to the CTS/Vc solution and vigorously stirred (500–600 rpm). The resultant CTS-SeNPs colloid (shown in Scheme 1) could be stable at room temperature for at least 4 weeks, without any visible precipitation. Bare SeNPs were gained by replacing CTS with deionized water during the synthesis of CTS-SeNPs.

Consequently, the SeNPs colloid was well mixed with setting amount of CTS solution. The new SeNPs/CTS solution was dropped into sodium citrate solution with moderate stirring. The cross-linking gelation took place and the resultant CTS/citrate gel containing SeNPs was washed with deionized water for several times. The gel was shredded and then spay-dried to obtain solid SeNPs-C/C. The Blank-C/C without any SeNP was prepared by replacing selenite with water. The Se content of each sample was measured by ICP-MS assay as described by Dufailly [38].

### Morphology observation and EDS detection

A TEM device (EM-2100; JEOL, Tokyo, Japan) was applied to study the morphological characteristics of SeNPs. Briefly, diluted SeNPs solution was dropped onto copper grid and dried in clean air, followed by TEM observation conducted at an accelerating voltage of 200 kV. For each sample, more than 20 TEM images were viewed and about 1500 SeNPs were studied to measure the size distribution. Besides, SEM was utilized to explore the details of SeNPs-C/C. The microparticles were coated with platinum in a vacuum state and the photographs were taken by SEM (S-4800; Hitachi, Tokyo, Japan) at an accelerating voltage of 2–10 kV. Meanwhile EDS was applied during TEM and SEM experiments.

### Measurement of size distribution

A particulate size analyzer [LS-POP(6); Zhuhai OMIC Instruments Co. Ltd., Zhuhai, People’s Republic of China] was utilized to determine the size distribution of SeNPs-C/C, with particle reflective index 1.70 and fluid reflective index 1.33. All measurements were conducted in triplicate.

### FTIR measurement

FTIR was used to measure changes in chemical structures of samples. Each sample was dried and then ground into homogeneous powders to record the IR spectra on a Nicolet Nexus 470 spectrometer (Thermo Fisher Scientific, Waltham, MA, USA). The spectra were acquired at 400–4000 cm^−1^ wavenumbers with a 4 cm^−1^ resolution.

### XPS determination

XPS analysis was performed by a high-resolution photoelectron spectrograph (Escalab 250Xi, Thermo Fisher Scientific) equipping a Monochromatic Al Kα x-ray source. A highly focused beam size of 500 μm × 500 μm was applied and the energy resolution was 0.05 eV. A dual beam charge neutralization system composed of an low-energy electron flood gun (~ 1 eV) and an argon ion gun (≤ 10 eV) was used.

### Observation of released SeNPs

50 mL of HCl or NaOH solution whose pH value was adjusted by HCl or NaOH to be a value ranging from 2 to 10, was mixed with 50 mg of SeNPs-C/C. The mixture was vigorously stirred at 37 °C for more than 8 h. At different time points, the released SeNPs in the mixture were investigated by TEM and EDS as previously described, after a filtration with Φ 0.8 μm Nylon filter.

### Measurement of storage stability

Two stability experiments including stress testing [45] and accelerated testing [46], were conducted. In the stress testing, SeNPs-C/C was presented at 60 ± 1 °C, 80 ± 5% RH and 5000 ± 500 Lx, respectively for 10 days. At the day “0”, “5” and “10”, the XPS patterns of SeNPs-C/C were recorded and the Se content of each sample was determined by ICP-MS assay [38]. In the accelerated testing, SeNPs-C/C was maintained under the condition of 40 ± 2 °C, 75 ± 5% RH, dark for at least 6 months. During the 6 months storage, the XPS patterns and Se content of SeNPs-C/C were recorded just after the 1st, 2nd, 3rd and 6th month.

### Acute lethal test in vivo

The acute toxicities of sodium selenite, Blank-C/C and SeNPs-C/C were measured by using KM mice and ICR mice as the model animal. After adaption for 3 days, 120 KM mice were randomly divided into 12 groups and 100 ICR mice were divided into 10 groups, with 10 mice each group. Among them, 7 groups of KM mice and 5 groups of ICR mice were used to test SeNPs-C/C while the remaining groups of mice were utilized to evaluate sodium selenite. Each sample was dissolved or dispersed in deionized water and then it was intragastrically administered at increasing doses. For SeNPs-C/C, the doses applied to KM mice and ICR mice were 6.60, 5.28, 4.22, 3.40, 2.70, 2.17, 1.73 and 15, 7.5, 3.75, 1.875, 0.9375 g kg^−1^ bw, respectively. For selenite, the doses for KM mice and ICR mice were 55, 38.5, 27, 18.8, 13.2 and 24.0, 12.9, 7.0, 3.7, 2.0 mg kg^−1^ bw, respectively. In another experiment, additional 30 KM mice and 20 ICR mice were treated with Blank-C/C at an accumulated dose of 20 and 15 g kg^−1^ bw, respectively, within 24 h to evaluate the LD_50_ of Blank-C/C. All mice were allowed free access to low-Se diet (< 0.1 mg Se kg^−1^ diet) and pure water after administration. Cumulative mortality within 14 days was recorded to calculate LD_50_ by Bliss assay [58].

### d-Gal-induced oxidative damage experiment

70 male KM mice, as shown in Table 3, were randomly divided into 5 groups: control group (saline, 10 mice), model group (d-gal, 15 mice) and three Se groups (d-gal + SeNPs-C/C, each 15 mice) comprising of L-Se, M-Se and H-Se. Mice in both model group and control group were daily administered sterile water by gavage. d-Gal was dissolved in 0.9% saline, and the aging model in Model and Se groups was established by daily subcutaneous injection of d-gal at a dose of 100 mg kg^−1^ bw around the neck and back for 45 days. In addition to the d-gal injection, mice in L-Se, M-Se and H-Se groups, were given 15.8, 31.6 and 62.3 mg kg^−1^ bw of SeNPs-C/C (dispersed in water) by intragastric administration, respectively. All mice were allowed free access to low-Se diet (< 0.1 mg Se kg^−1^ diet) and water.

During the experiment, the body weight of mice was recorded. After the last treatment, mice were sacrificed to obtain serum, liver, spleen and thymus to measure the organ index (relative organ weight) according to Zheng [30]. The serum was used to determined the levels of TBARS, GSH, CAT, SOD and GSH-Px, following the instructions of commercial kits. Liver was fixed in 10% buffered formaldehyde, embedded in paraffin, sectioned, and stained with hematoxylin and eosin (HE) dye [3, 51] for observation.

### Statistical analysis

In all the experiments, data were presented as mean ± SD. The Student’s *t* test was utilized to examine the differences between the groups by using SPSS software program (version 17.0 for Windows). A *P* value of < 0.05 was considered statistically significant.

## Abbreviations

Blank-C/C: blank chitosan/citrate complex
BSA: bovine serum albumin
bw: body weight
CAT: catalase
CTS: chitosan
d-gal: d-galactose
EDS: energy-dispersive X-ray spectroscopy
FTIR: Fourier transform infrared spectroscopy
GSH: glutathione
GSH-Px: glutathione peroxidase
HE: hematoxylin and eosin
ICR: Institute of Cancer Research
ICP-MS: inductively coupled plasma mass spectrometry
KM: Kunming
LD_50_: median lethal dose
OFR: oxygen-derived free radicals
RH: relative humidity
ROS: radical oxygen species
Se: selenium
SEM: scanning electron microscopy
SeNPs: selenium nanoparticles
SeNPs-C/C: selenium nanoparticles-loaded chitosan/citrate complex
SOD: superoxide dismutase
SPF: specific-pathogen-free
TBARS: thiobarbituric acid-reactive substances
TEM: transmission electron microscopy
Vc: ascorbic acid
XPS: X-ray photoelectron spectroscopy
